# Combined Plant Growth-Promoting Bacteria Inoculants Were More Beneficial than Single Agents for Plant Growth and Cd Phytoextraction of *Brassica juncea* L. during Field Application

**DOI:** 10.3390/toxics10070396

**Published:** 2022-07-17

**Authors:** Qiong Wang, Shun’an Xu, Zheyu Wen, Qizhen Liu, Lukuan Huang, Guosheng Shao, Ying Feng, Xiaoe Yang

**Affiliations:** 1 MOE Key Laboratory of Environment Remediation and Ecological Health, College of Environmental and Resource Sciences, Zhejiang University, Hangzhou 310058, China; wq122506@zju.edu.cn (Q.W.); 22014150@zju.edu.cn (S.X.); wzywenzheyu@zju.edu.cn (Z.W.); 12214012@zju.edu.cn (Q.L.); 12114025@zju.edu.cn (L.H.); xeyang@zju.edu.cn (X.Y.); 2 College of Ecology, Taiyuan University of Technology, Taiyuan 030024, China; 3 State Key Laboratory of Rice Biology, China National Rice Research Institute, Hangzhou 310006, China

**Keywords:** cadmium, oilseed rape, plant–microbe interaction, phytoremediation, Cd removal

## Abstract

Single or combined plant growth-promoting bacteria (PGPB) strains were widely applied as microbial agents in cadmium (Cd) phytoextraction since they could promote plant growth and facilitate Cd uptake. However, the distinct functional effects between single and combined inoculants have not yet been elucidated. In this study, a field experiment was conducted with single, double and triple inoculants to clarify their divergent impacts on plant growth, Cd uptake and accumulation at different growth stages of *Brassica juncea* L. by three different PGPB strains (*Cupriavidus* SaCR1, *Burkholdria* SaMR10 and *Sphingomonas* SaMR12). The results show that SaCR1 + SaMR10 + SaMR12 combined inoculants were more effective for growth promotion at the bud stage, flowering stage, and mature stage. Single/combined PGPB agents of SaMR12 and SaMR10 were more efficient for Cd uptake promotion. In addition, SaMR10 + SaMR12 combined the inoculants greatly facilitated Cd uptake and accumulation in shoots, and enhanced the straw Cd extraction rates by 156%. Therefore, it is concluded that the application of PGPB inoculants elevated Cd phytoextraction efficiency, and the combined inoculants were more conductive than single inoculants. These results enriched the existing understanding of PGPB agents and provided technical support for the further exploration of PGPB interacting mechanisms strains on plant growth and Cd phytoextraction, which helped establish an efficient plant–microbe combined phytoremediation system and augment the phytoextraction efficiency in Cd-contaminated farmlands.

## 1. Introduction

Phytoremediation technology is an environmentally friendly, simple and effective strategy for alleviating and cleaning up the soil contaminants of multiple heavy metals including cadmium (Cd), lead (Pb), chromium (Cr), arsenic (As), etc [1]. More and more studies have shown that *Brassica* species have great potential for phytoremediation since they could tolerate and accumulate high amounts of heavy metals such as Cd [2]. The relevant mechanisms for high Cd tolerance were also meticulously elucidated, including an elevated antioxidant defense system, organic acid secretion, the augmentation of total amino acid production, chelation, and Cd compartmentation into the cell wall [1,3,4,5]. Therefore, species such as *B. rapa* and *B. juncea* are suitable for Cd phytoremediation as they tolerate and accumulate relatively high levels of Cd [6,7].

Plant growth-promoting bacteria (PGPB) were verified as beneficial to plant growth, Cd uptake and Cd accumulation as well [8,9]. For example, the inoculation of *Pseudomonas* Sasm05 promoted plant biomass by 45% and Cd uptake by 59% in *Sedum alfredii* Hance [10]. The inoculation of *Bacillus* QX8 and QX13 enhanced the dry weight of *Solanum nigrum* L. with 136–170% in shoots and 142–196% in roots, and Cd concentrations were also increased to 228–281%, respectively [11]. A previous study also revealed that *Sphingomonas* SaMR12 promotes biomass in *B. juncea* up to 71% in shoots and 81% in roots, and enhanced Cd accumulation up to 172% under Cd stress [9]. Apart from the beneficial characteristics of PGPB strains, the interactions between different strains, and the antagonistic relationships of PGPB and soil inherent microorganisms could also influence plant growth and Cd uptake [12,13]. It is reported that the combined inoculation of *Ncr-1* and *Ncr-8* was effective in the plant growth promotion and nickel translocation of their host plant *N. caerulescens* [14]. The triple inoculation of *Azospirillum brasilense*, *Burkholderia cepacian,* and *Enterobacter cloacae* increased the biomass of strawberries [15]. The triple inoculation of PGPB consortia with different ecological niches was also verified to be effective to promote plant growth and Cd uptake in *B. juncea* [16]. However, the distinct effects between single PGPB inoculants and combined PGPB inoculants on promoting plant growth and Cd uptake remain unclear.

Therefore, three PGPB strains were adapted to Cd-contaminated soil for a field experiment to elucidate the differences among single PGPB inoculants, double PGPB inoculants, and triple PGPB inoculants in plant growth, plant Cd uptake and accumulation, Cd phytoextraction efficiency, and soil Cd removal rates. All strains used in this study were isolated from hyperaccumulator *S. alfredii*, and were verified as beneficial for plant growth and the Cd uptake of *B. juncea* in our previous study [17]. The objective of this study was to determine the effective PGPB inoculants for facilitating both plant growth and Cd phytoremediation. This study will expound on the rule of efficient PGPB consortia for effective Cd removal and sustainable crop production in the agricultural field.

## 2. Materials and Methods

### 2.1. Preparation of Bacterial Suspensions

Three bacterial strains, namely *Cupriavidus* SaCR1, *Burkholdria* SaMR10, and *Sphingomonas* SaMR12 were selected according to their plant growth-promoting (PGP) traits on plant growth and the Cd uptake of *B. juncea* [17]. Detailed PGP traits and the 16 S rDNA accession number were listed in Table 1. Bacterial strains were cultured in Luria–Bertani (LB) broth medium at 30 °C for 24 h with continuous shaking. Cells were washed afterward, followed by suspension and then adjusted the optical density (OD) from 600 nm to 1.0 [9].

### 2.2. Field Layout and Experimental Design

Eight treatments were set in this experiment: (1) non-inoculated as control; (2) SaCR1 single inoculants; (3) SaMR10 single inoculants; (4) SaMR12 single inoculants; (5) SaCR1 + SaMR10 combined inoculants; (6) SaCR1 + SaMR12 combined inoculants; (7) SaMR10 + SaMR12 combined inoculants; and (8) SaCR1 + SaMR10 + SaMR12 combined inoculants. After culturing for 24 h, the bacterial suspensions were obtained with centrifugation to OD_600nm_ = 1.0. Combined inoculants were prepared with the same volume of bacterial suspensions of the corresponding individual components (1:1 and 1:1:1 in double and triple combined inoculants). Equivalent sterilized water was added in the non-inoculated treatment.

**Figure 1:**
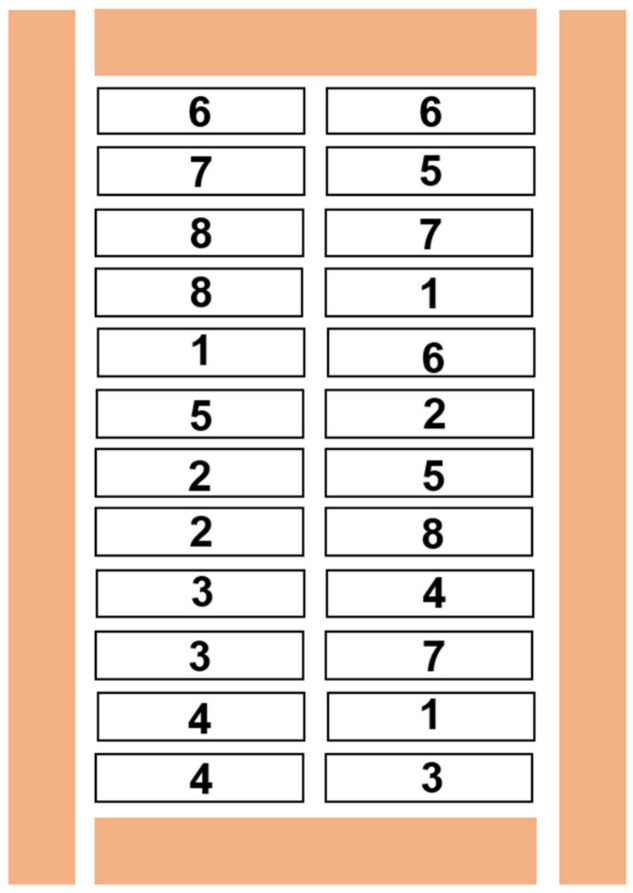
Diagrammatic field layout of the experiment. The field was divided into 24 plots, each 12 m in length and 1.55 m in width. The interval between each plot is 20 cm, and guard raw were set around the experimental plots (the orange cases). Numbers 1–8 represent the different treatments, and the same numbers represent three replicates of the experimental treatment.

The field experiment was located in the experimental field of the China National Rice Research Institute in Fuyang District, Hangzhou. The climate of Fuyang belongs to the subtropical monsoon climate, and its basic characteristics are cold winter and hot summer, with four distinct seasons. There are also abundant precipitation and sufficient sunshine in spring, synchronous rain and heat in summer, and complementary light and temperature in autumn and winter. All these climate characteristics were suitable for the cultivation and growth of oilseed rape. The experimental block is a paddy-upland rotation paddy field, with a soil pH 6.3; soil organic matter 28.6 mg·kg^−1^; total nitrogen content 1.88 g·kg^−1^; CEC content 15.6 cmol·kg^−1^; soil total Cd content 1.49 mg·kg^−1^; soil available Cd content 0.72 mg·kg^−1^. Before plant transplanting, 750 kg·ha^−1^ compound fertilizer was applied into the field as a basal fertilizer, in which the nitrogen: phosphate: potassium = 15:14:16. None of the extra nutrients (e.g., NPK fertilizer) were added to the experimental duration. After the rice harvest in October, straws were removed and the field was divided into 24 equal plots. Bacterial suspensions were sprayed into each plot with an equivalent volume of 60 mL based on our previous study [16]. The diagrammatic field layout is depicted in Figure 1.

### 2.3. Determination of Cd Contents in Plant Samples and Soil Samples

Samples were collected at different growth stages, including the seedling stage, bud stage, flowering stage, and the mature stage. The harvested plant samples were oven-dried at 65 °C until constant weights were reached. After recording the dry weights, plant samples were ground into powder. Then, 0.2 g samples were used for digestion with HNO_3_ and H_2_O_2_ (5:1, *v*/*v*) solution until clear, then Cd contents were determined using an Inductively Coupled Plasma Mass Spectrometer (ICP-MS, PlasmaQuant^®^ MS, Analytik Jena, Jena, Germany). Quality control was processed with National Reference Materials (GBW (E) 100495, plant Cd contents 0.36 ± 0.02 mg·kg^−1^).

The soil samples were collected at the mature stage. After being naturally dried and ground to pass through a 2 mm sieve, the total Cd contents in the soil were measured by digestion with HNO_3_:HClO_4_: HF = 5:1:1 (*v*/*v*/*v*) and determined by an ICP-MS (PlasmaQuant^®^ MS, Analytik Jena, Jena, Germany). National Reference Materials (GBW07917, soil Cd contents 0.62 ± 0.04 mg·kg^−1^) were used for quality control.

### 2.4. Calculation of Plant Cd Extraction and Soil Cd Removal

After sample harvesting at the mature stage, plant Cd extraction, plant Cd extraction rates, soil Cd removal, and soil Cd removal rates were calculated according to [18] using the following formulas:

Cd extraction by straw (mg·plot^−1^) = Cd contents in straw (mg·kg^−1^) × straw yield (kg·plot^−1^)

Cd extraction by seeds (mg·plot^−1^) = Cd contents in seeds (mg·kg^−1^) × seeds yield (kg·plot^−1^)

Cd extraction rates by straw (%) = Cd extraction by straw/initial Cd accumulation in soil × 100

Cd extraction rates by seeds (%) = Cd extraction by seeds/initial Cd accumulation in soil × 100

Soil Cd removal (mg·plot^−1^) = initial Cd accumulation in soil (mg·plot^−1^) − [soil Cd contents in mature stage (mg·kg^−1^) × plot soil biomass (kg·plot^−1^)]

Soil Cd removal rates (%) = soil Cd removal/initial Cd accumulation in soil × 100

Initial Cd accumulation in soil (mg·plot^−1^) = initial soil Cd concentration (mg·kg^−1^) × plot soil biomass (kg·plot^−1^)/1000

Plot soil biomass (kg·plot^−1^) = plot length (m) × plot width (m) × soil depth (m) × bulk density (g·cm^−3^)

Here, the plot length is 12 m, the plot width is 1.55 m, soil depth is 20 cm, and the bulk density is 1.1 g·cm^−3^.

### 2.5. Data Analysis

The data analysis of this study was performed using the SPSS statistical 20.0 software (SPSS Inc., Chicago, IL, USA). One-way analysis of variance (ANOVA) proceeded at a significance of *p* < 0.05 indicated by Duncan’s test. Graphical work was conducted with Origin 8.0 (Northampton, MA 01060, USA).

## 3. Results

### 3.1. Effects of PGPB Inoculants on Plant Growth

**Figure 2:**
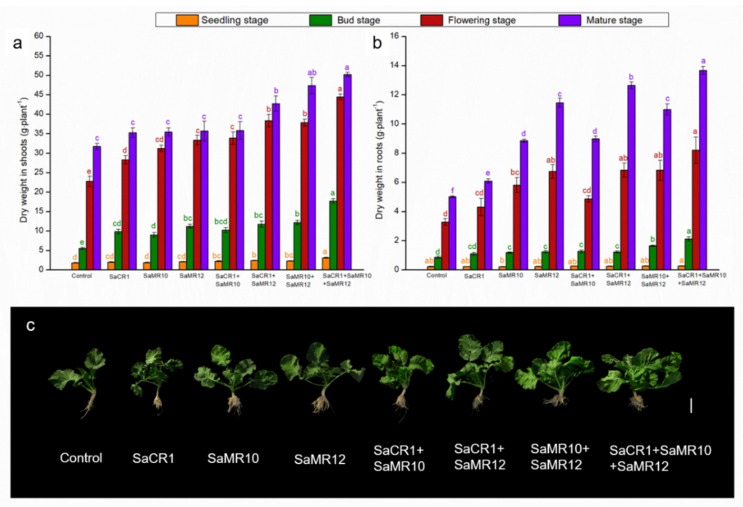
Effects of PGPB inoculants on dry weight (DW) in (**a**) shoots and (**b**) roots of *B. juncea* at different growth stages. Orange, green, red, and purple represent the seedling stage, bud stage, flowering stage, and mature stage, respectively. The letters of corresponding colors indicate the significant differences among different treatments at the same growth stage. (**c**) Plant growth condition at the bud stage. Bar = 10 cm.

The dry weights (DW) in shoots at the seedling stage were increased by 4.5–70.2% after adopting PGPB inoculants, among which SaCR1 + SaMR10 + SaMR12 combined inoculants showed the largest augmentation in shoot biomass promotion. In addition, combined inoculants were more beneficial than single inoculants to facilitate shoot DW at the seedling stage. PGPB inoculants enhanced shoot DW by 63.1–220.1% and 24.4–95.2% at the bud stage and flowering stage, respectively (Figure 2a). Combined inoculants of SaCR1 + SaMR10 + SaMR12 were most efficient for shoot promotion at the bud stage (Figure 2a,c). At the mature stage, SaCR1 + SaMR12 combined inoculants, SaMR10 + SaMR12 combined inoculants, and SaCR1 + SaMR10 + SaMR12 combined inoculants significantly increased shoot DW by 34.6%, 49.1%, and 58.2%, respectively (Figure 2a). In summary, SaCR1 + SaMR10 + SaMR12 combined inoculants were the most effective agents for shoot biomass promotion in all plant growth stages (Figure 2a).

However, PGPB inoculants induced inconspicuous differences in plant root DW at the seedling stage (Figure 2b). At the bud stage, root DW was significantly enhanced with PGPB inoculants, among which SaCR1 + SaMR10 + SaMR12 combined inoculants were most effective with 146.7% augmentation on root biomass (Figure 2b,c). Compared with non-inoculated treatment, PGPB inoculants distinctly enhanced root DW at the flowering stage by 31.2–149.7%. At the mature stage, PGPB inoculants significantly facilitated root DW with 21.6–172.7% (Figure 2b). Similarly, the SaCR1 + SaMR10 + SaMR12 combined inoculants were most beneficial to root biomass promotion in all plant growth stages (Figure 2b).

### 3.2. Effects of PGPB Inoculants on Seed Yield, Seed Cd Uptake, and Accumulation

**Figure 3:**
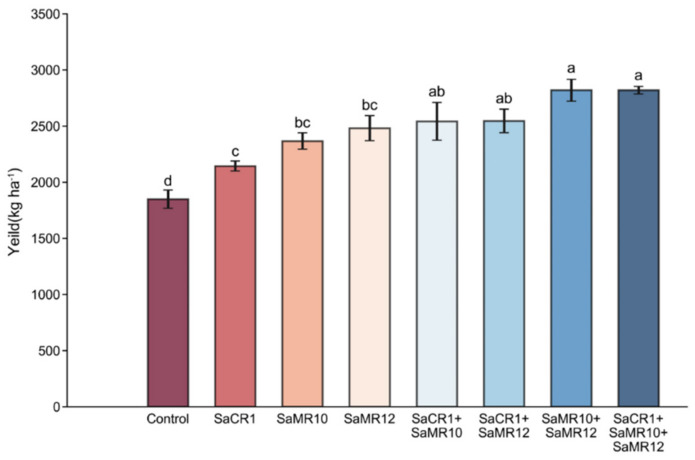
Effects of PGPB inoculants on the seed yield of *B. juncea*. The letters above the column indicate the significant differences among different treatments at *p* < 0.05.

Compared with non-inoculated treatment, PGPB inoculants significantly facilitated the seed yield by 15.9–52.5%. Specifically, combined PGPB inoculants were more effective than single inoculants (Figure 3).

**Figure 4:**
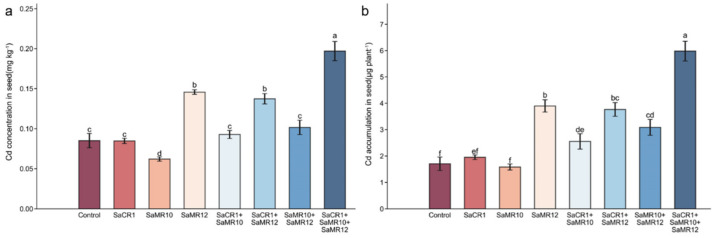
Effects of PGPB inoculants on the seed (**a**) Cd concentration and (**b**) Cd accumulation. The letters above the column indicate the significant differences among different treatments at *p* < 0.05.

Compared with non-inoculated treatment, SaMR10 single inoculants significantly decreased the seed Cd concentration by 27.0%, while SaMR12 single inoculants, SaCR1 + SaMR12 combined inoculants, and SaCR1 + SaMR10 + SaMR12 combined inoculants all promoted seed Cd concentration at a significant level by 71.2%, 61.3%, and 131.5% accordingly (Figure 4a). Additionally, the seed Cd accumulation was slightly decreased with SaMR10 single inoculants, while SaMR12 single inoculants and SaCR1 + SaMR10 + SaMR12 combined inoculants significantly enhanced the seed Cd accumulation by 128.2% and 250.2%, respectively (Figure 4b).

### 3.3. Effects of PGPB Inoculants on Plant Cd Uptake and Accumulation

**Figure 5:**
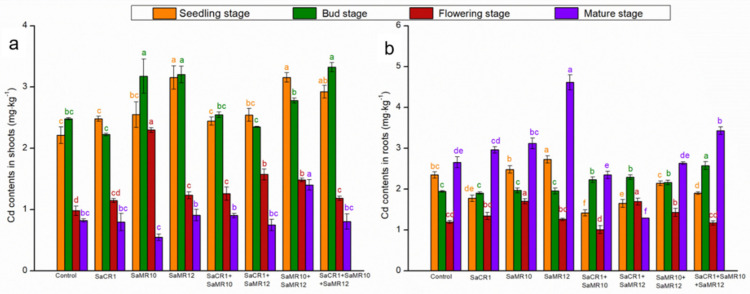
Effects of PGPB inoculants on Cd contents in (**a**) shoots and (**b**) roots of *B. juncea* at different growth stages. Orange, green, red, and purple represent the seedling stage, bud stage, flowering stage, and mature stage, respectively. The letters of corresponding colors indicate the significant differences among different treatments at the same growth stage.

Cd contents in shoots were increased from seedling stage to bud stage, but subsequently declined (Figure 5). At the seedling stage, SaMR12 single inoculants, SaMR10 + SaMR12 combined inoculants, and SaCR1 + SaMR10 + SaMR12 combined inoculants significantly enhanced the shoot Cd contents by 42.7%, 42.7%, and 32.1% accordingly. At the bud stage, SaMR10 single inoculants, SaMR12 single inoculants, and SaCR1 + SaMR10 + SaMR12 combined inoculants significantly enhanced shoot Cd contents by 28.1%, 29.2%, and 34.0% accordingly. Shoot Cd contents during the flowering stage were generally lower than the seedling stage and bud stage, and PGPB inoculants improved the Cd contents by 17.0–134.4%. At the mature stage, only SaMR10 + SaMR12 combined inoculants significantly enhanced Cd contents by 70.5% (Figure 5).

**Figure 6:**
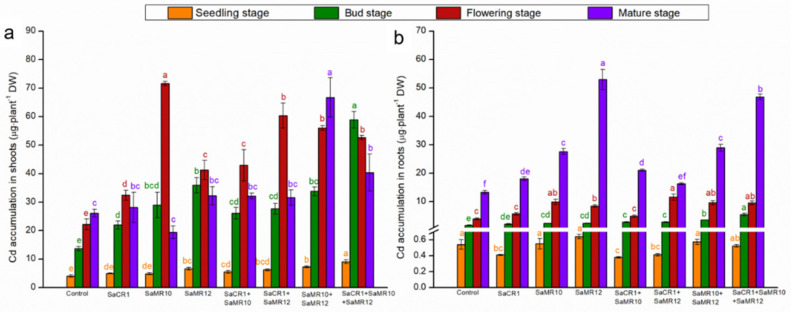
Effects of PGPB inoculants on Cd accumulation in (**a**) shoots and (**b**) roots of *B. juncea* at different growth stages. Orange, green, red, and purple represent the seedling stage, bud stage, flowering stage, and mature stage, respectively. The letters of corresponding colors indicate the significant differences among different treatments at the same growth stage.

The Cd contents in roots were firstly decreased from the seedling stage to the flowering stage, which then increased in the mature stage (Figure 6). At the seedling stage, SaMR10 single inoculants and SaMR12 single inoculants augmented the root Cd contents by 5.7% and 16.2%, respectively. Instead, SaCR1 single inoculants, SaCR1 + SaMR10 combined inoculants, SaCR1 + SaMR12 combined inoculants, and SaCR1 + SaMR10 + SaMR12 combined inoculants significantly decreased the root Cd contents by 24.4%, 39.6%, 29.6%, and 19.0%, respectively. PGPB combined inoculants were more effective for elevating root Cd contents than single inoculants at the bug stage since SaCR1 + SaMR10 combined inoculants, SaCR1 + SaMR12 combined inoculants, SaMR10 + SaMR12 combined inoculants, and SaCR1 + SaMR10 + SaMR12 combined inoculants significantly increased root Cd contents by 11.3–32.4%. At the flowering stage, SaMR10 single inoculants, SaCR1 + SaMR12 combined inoculants, and SaMR10 + SaMR12 combined inoculants significantly enhanced the root Cd contents by 42.7%, 41.9%, and 19.8%, respectively. At the mature stage, SaMR10 single inoculants, SaMR12 single inoculants, and SaCR1 + SaMR10 + SaMR12 combined inoculants significantly improved root Cd contents by 17.5%, 73.9%, and 29.2%, respectively (Figure 6).

### 3.4. Effects of PGPB Inoculants on Cd Translocation Factor

**Figure 7:**
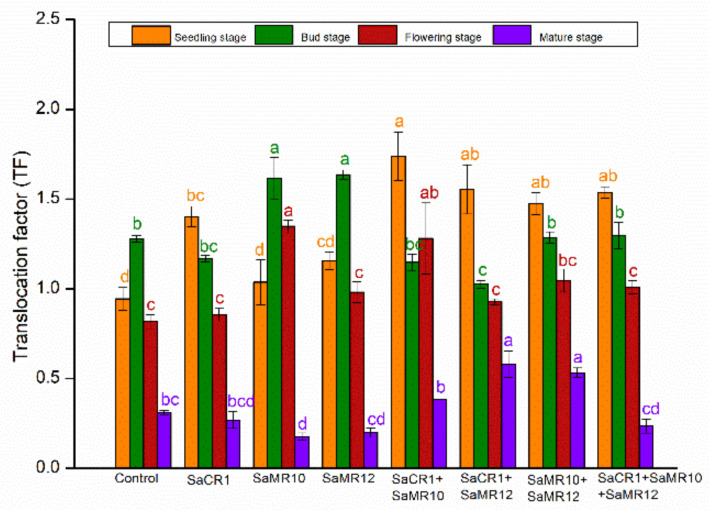
Effects of PGPB inoculants on the translocation factor of *B. juncea* at different growth stages. Orange, green, red, and purple represent the seedling stage, bud stage, flowering stage, and mature stage, respectively. The letters of corresponding colors indicate the significant differences among different treatments at the same growth stage.

Cd translocation factor (TF) was calculated by the formula: Cd translocation factor (TF) = Cd contents in shoots (mg·kg^−1^)/Cd contents in roots (mg·kg^−1^). At the seedling stage, Cd TF was lower than 1.0 in the non-inoculated control, and PGPB inoculants enhanced Cd TF with 9.8–84.0% among which SaCR1 + SaMR10 combined inoculants were more effective than other treatments. At the bud stage, Cd TF were higher than 1.0 in all treatments. Compared with non-inoculated control, only SaMR10 single inoculants and SaMR12 single inoculants significantly improved Cd TF by 26.4% and 28.0%, respectively. At the flowering stage, Cd TF was lower than 1.0 in the non-inoculated control, SaMR10 single inoculants, and SaCR1 + SaMR10 combined inoculants significantly promoted Cd TF by 64.8% and 56.7%, respectively. Cd TF at the mature stage were greatly reduced to <1.0. Compared with non-inoculated control, only SaCR1 + SaMR10 combined inoculants and SaMR10 + SaMR12 combined inoculants significantly increased Cd TF with 86.7% and 71.2%, respectively (Figure 7).

### 3.5. Effects of PGPB Inoculants on Plant Cd Extraction and Soil Cd Removal

Compared with non-inoculated control, most PGPB inoculants (except SaMR10 single inoculants) facilitated Cd extraction by straw, among which SaMR10 + SaMR12 combined inoculants were the most efficient with a 156.4% augmentation. Additionally, Cd extraction rates by straw with SaMR10 + SaMR12 combined inoculants was 3.66%, which is 256.0% higher than that in the non-inoculated control. Compared with straw, Cd extraction by seeds was a slight proportion with less than 14% of total Cd extraction by shoots. PGPB inoculants significantly promoted Cd extraction in seeds by 49.6–250.2%. With SaCR1 + SaMR10 + SaMR12 combined inoculants, Cd extraction rates by seeds reached 0.33%, which is 266.7% higher than that in the non-inoculated control. In addition, PGPB inoculants greatly enhanced soil Cd removal by 19.9–70.9%, and soil Cd removal rates were also promoted with PGPB inoculants (Table 2).

## 4. Discussion

This study investigated the distinctive effects between single inoculants and combined inoculants on plant growth, Cd uptake and accumulation, and the Cd phytoremediation efficiency of *B. juncea* at different growth stages. These results provide theoretical evidence and lay a practical foundation for evaluating the impacts of PGPB inoculants as microbial agents during Cd phytoextraction in field application.

### 4.1. PGPB Combined Inoculants Were More Beneficial to Plant Growth

PGPB strains are widely distributed in the plant rhizosphere and endosphere, playing important roles in plant growth conditions in contaminated soil [19,20]. For example, inoculation of *Burkholderia contaminans ZCC* significantly promoted soybean growth by 23.96%, 78.18% in shoots and 20.14%, 28.81% in roots under 0.5 μM and 2.5 μM Cd^2+^ treatment, respectively [21]. Both single and combined inoculating wheat with *Citrobacter werkmanii* strain WWN1 and *Enterobacter cloacae* strain JWM6 improved the dry weight by 65–179% [22]. In this study, single and combined inoculants all promoted plant growth at different growth stages, suggesting that PGPB inoculants could facilitate the growth condition of oilseed rape in Cd-contaminated soil (Figure 2). PGPB inoculants promoted shoot biomass at the seedling stage, while hardly affecting root biomass, which may account for the thin and weak root development at the seedling stage (Figure 2). At the bud stage, flowering stage, and mature stage, PGPB inoculants augmented the plant growth, among which SaCR1 + SaMR10 + SaMR12 combined inoculants were more effective than single inoculants (Figure 2), indicating that combined PGPB inoculants were more beneficial to plant biomass accumulation during the middle and late growth stages. Moreover, combined inoculants were superior to seed yield (Figure 3). Such beneficial effects could be attributed to ACC deaminase activity, IAA production, siderophore production, and the phosphate solubilization of PGPB strains (Table 1) [23]. On the other hand, PGPB could regulate the physiological processes of plants and reduce the stress from heavy metals on plants during the combined plant–microbe remediation of heavy metal pollution in soil [24]. In addition, PGPB could facilitate plant growth under Cd stress by activating antioxidative enzymes and the glutathione–ascorbic acid cycle [9], regulating hormonal and nutritional balance [25], and protecting against pathogenic microorganisms and herbivores [26]. Thus, our results show that combined inoculants were more suitable as remediating agents for plant biomass augmentation during Cd phytoremediation process in field application.

### 4.2. PGPB Combined Inoculants Were More Pronounced for Cd Phytoremediation

Apart from biomass augmentation, PGPB–plant interaction also facilitated Cd uptake and accumulation [19,24]. The interaction effects between different strains of PGPB inoculants may change the capability of heavy metals absorption, thus leading to the differential Cd uptake and accumulation by plants. A synthetic community constructed with beneficial microorganisms as well as PGPB strains was inoculated to willow, and the results showed that the impacts of different consortia towards Cd uptake in willow were greatly different [27]. In this study, SaMR10 single inoculants, SaMR12 single inoculants and SaMR10 + SaMR12 combined inoculants were more conducive to plant Cd uptake and accumulation at different growth stages (Figure 5 and Figure 6), which could be explained by the better performance of SaMR10 and SaMR12 single inoculation in comparison of SaCR1 in facilitating the Cd uptake of *B. juncea* [17]. It was reported that the application of multiple PGPB strains was more productive to plant growth promotion and heavy metal accumulation [23,28], which also supported our results. In addition, the results showed that the Cd contents in seeds were below the thresholds of the China National Food Safety Standards [29] (GB 2762-2017). Considering the Cd threat to human health, the seeds were usually used for industrial oil extraction. Additionally, straws after phytoextraction were removed from the field and used for pyrolytic carbons [30], nanoporous carbons [31], and biochar [27] as well. Cd concentration in shoots at mature stage declined but increased in roots (Figure 5), indicating the consistency of decreased Cd TF at mature stage (Figure 7). With SaMR10 single inoculants, Cd uptake and accumulation at the mature stage in seeds were reduced due to the decreased Cd TF and Cd concentration in shoots (Figure 4, Figure 5, Figure 6 and Figure 7). Furthermore, our results suggest that SaMR10 + SaMR12 combined inoculants were more suitable for elevated plant Cd extraction and soil Cd removal (Table 2). In summary, the co-application of *B. juncea* and combined inoculants were recommended to be productive combinations to facilitate plant growth and Cd uptake, thus contributing to augmented Cd phytoextraction and safe agricultural production.

## 5. Conclusions

The results concluded that PGPB inoculants were effective for plant growth promotion and seed yield production, in which SaCR1 + SaMR10 + SaMR12 combined inoculants were more conducive. Moreover, SaMR10 + SaMR12 combined inoculants showed more augmented impacts on Cd uptake and accumulation in straw, which enhanced Cd extraction rates in straw by 156.0%. In conclusion, the application of PGPB inoculants was beneficial for facilitating both plant growth and the Cd phytoremediation of *B. juncea*, and combined inoculants were more effective than single inoculants. These results amplified the present knowledge about PGPB co-inoculation and provided theoretical guidance for the application of PGPB inoculants as bioaugmentation agents in Cd-contaminated fields to further guarantee sustainable agriculture development and safe production.

## Figures and Tables

**Table 1:** Detailed information on bacterial strains used in this study.

	Genus Affiliation	16S rDNA Accession No.	ACC Deaminase Activity	IAA Production	Siderophore Production	Phosphate Solubilization
SaCR1	*Cupriavidus*	JQ806419	−	+	+	+
SaMR10	*Burkholdria*	HQ730964	+	+	+	+
SaMR12	*Sphingomonas*	JN573357	−	+	+	+

All bacterial strains were isolated from *Sedum alfredii* Hance. +: positive; −: negative; ACC, 1-aminocyclop ropane-1-carboxylate; IAA, indoleacetic acid.

**Table 2:** Effects of PGPB inoculants on plant Cd extraction and soil Cd removal. The letters ^a–f^ represent the significant level among different treatment at *p* < 0.05.

Treatments	Cd Extraction by Straw (mg·plot^−1^)	Cd Extraction Rates by Straw (%)	Cd Extraction by Seeds (mg·plot^−1^)	Cd Extraction Rates by Seeds (%)	Soil Cd Removal (mg·plot^−1^)	Soil Cd Removal Rates (%)
Control	87.50 ± 4.42 ^c^	1.43	5.72 ± 0.84 ^f^	0.09	1196.29 ± 89.51 ^b^	19.61
SaCR1	94.31 ± 17.72 ^bc^	1.55	6.54 ± 0.29 ^ef^	0.11	1983.93 ± 411.81 ^ab^	32.52
SaMR10	65.05 ± 7.38 ^c^	1.07	5.31 ± 0.38 ^f^	0.09	1578.86 ± 115.23 ^ab^	25.88
SaMR12	108.03 ± 10.55 ^bc^	1.77	13.05 ± 0.78 ^b^	0.21	1843.53 ± 293.25 ^ab^	30.22
SaCR1 + SaMR10	107.73 ± 3.19 ^bc^	1.77	8.56 ± 0.98 ^de^	0.14	2044.72 ± 168.71 ^a^	33.52
SaCR1 + SaMR12	105.89 ± 8.88 ^bc^	1.74	12.61 ± 0.87 ^bc^	0.21	1820.83 ± 163.36 ^ab^	29.85
SaMR10 + SaMR12	223.45 ± 23.09 ^a^	3.66	10.34 ± 1.00 ^cd^	0.17	1434.56 ± 149.60 ^ab^	23.52
SaCR1 + SaMR10 + SaMR12	135.23 ± 21.58 ^b^	2.22	20.03 ± 1.26 ^a^	0.33	1920.34 ± 295.75 ^ab^	31.48

## Data Availability

The data presented in this study are available on request from the corresponding author.
